# Supplementary material for: The Onset of Subtalar Joint Monoarthritis in a Patient with Rheumatoid Arthritis
Source: Diagnostics (Basel). 2022 Sep 25;12(10):2311. doi: 10.3390/diagnostics12102311 (PMC9600197; doi:10.3390/diagnostics12102311)
Supplement: Supplementary file 1 [file diagnostics-12-02311-s001.zip › diagnostics-1907344-supplementary.pdf]

diagnostics

Case Report

The Onset of Subtalar Joint Monoarthritis in a Patient with Rheumatoid Arthritis

Hiroki Wakabayashi *,†, Kenta Nakata †, Akinobu Nishimura †, Masahiro Hasegawa and Akihiro Sudo

Department of Orthopedic Surgery, Mie University Graduate School of Medicine, Tsu 514-8507, Japan
* Correspondence: whiroki@clin.medic.mie-u.ac.jp; Tel.: +81-59-231-5022
† These authors contributed equally to this work.

Supplementary Materials

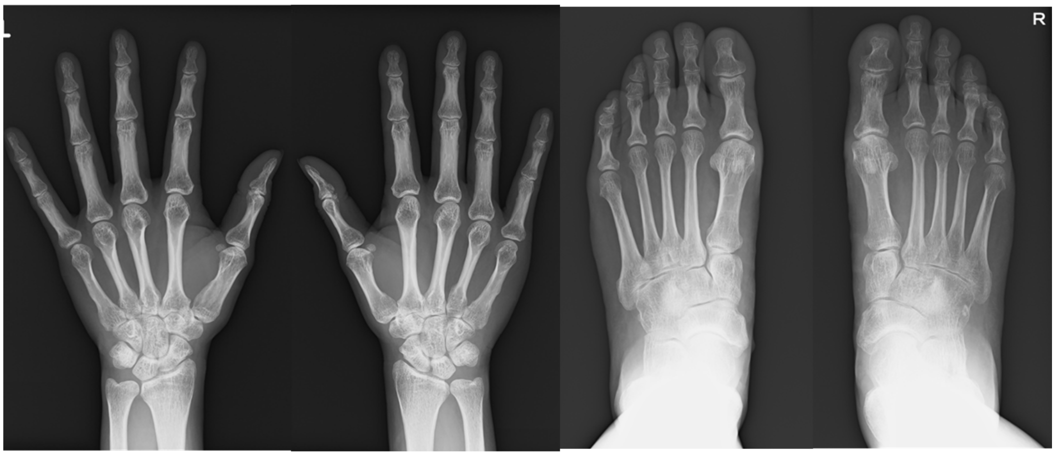

(**a**) (**b**)

Figure S1. The plain radiographs of her hands (**a**) and foot (**b**).
